# Movement Patterns in a Partial Migrant: A Multi-Event Capture-Recapture Approach

**DOI:** 10.1371/journal.pone.0096478

**Published:** 2014-05-06

**Authors:** Marie-Lucile Gourlay-Larour, Roger Pradel, Matthieu Guillemain, Jean-Sébastien Guitton, Monique L'Hostis, Hugues Santin-Janin, Alain Caizergues

**Affiliations:** 1 Office National de la Chasse et de la Faune Sauvage, Nantes, France; 2 Université Nantes Angers Le Mans, Nantes, France; 3 Centre d'Ecologie Evolutive et Fonctionnelle UMR 5175, CNRS, Montpellier, France; 4 Office National de la Chasse et de la Faune Sauvage, Arles, France; 5 Ecole Nationale Vétérinaire Agroalimentaire et de l'Alimentation Nantes-Atlantique, Oniris, Nantes, France; 6 Office National de la Chasse et de la Faune Sauvage, Le Perray-en-Yvelines, France; Texas Christian University, United States of America

## Abstract

Partial migration is a pervasive albeit poorly studied phenomenon by which some individuals of a population migrate while others are residents. It has tremendous consequences on seasonal variations of population size/structure and therefore management. Using a multi-event capture-mark-recapture/recovery (CMR) approach, we assessed seasonal site occupancy, survival and site fidelity of a partially migratory diving duck, the Common pochard (*Aythya ferina*), in an area potentially including both local breeders and winter visitors. The modelling exercise indeed discriminated two different categories of individuals. First, locally breeding females which had a probability of being present in our study area during winter of 0.41. Females of this category were found to be more faithful to their breeding site than males (breeding site fidelity probabilities of 1 and 0.11, respectively). The second category of birds were winter visitors, which included adults of both sexes, whose probability of being present in the study area during the breeding season was nil, and young of both sexes with a 0.11 probability of being present in the area during the breeding season. All wintering individuals, among which there was virtually no locally breeding male, displayed a high fidelity to our study area from one winter to the next (0.41–0.43). Estimated annual survival rates differed according to age (adults 0.69, young 0.56). For both age classes mortality was higher during late winter/early spring than during summer/early winter. Our study is among the first to show how and under which conditions the multi-event approach can be employed for investigating complex movement patterns encountered in partial migrants, providing a convenient tool for overcoming state uncertainty. It also shows why studying patterns of probability of individual presence/movements in partial migrants is a key towards understanding seasonal variations in numbers.

## Introduction

Measuring the extent to which individuals move and are faithful to their seasonal ranges is key to understanding the structure and dynamics of migratory bird populations [1], [2]. Studying migration is also essential from a management perspective [3], [4] and is urgently needed owing to multiple anthropogenic stressors (habitat destruction, climate warming, hunting…) affecting some migratory species [1], [3]. Birds show a great diversity of migratory patterns and strategies, both among and within species [3]. Partial migration, in which migrants and residents coexist in the same population, is the most common type of migration from which all other migration types can be derived [5]. In this system, coexistence of migration tactics can occur either on the breeding ground when migrants and residents breed in sympatry but overwinter apart from each other (“non-breeding partial migration”, [6]), or on the wintering grounds when they overwinter together but breed in allopatry (“breeding partial migration”, [7]). Many widely distributed bird species of the Holarctic region may be strict migrants in the northern part of their breeding range, residents in the southern part, and partial migrants in between [3], [8]. In such species, the wintering area of strict migratory populations overlaps with the breeding ranges of partially migratory populations, which may include a more or less significant proportion of year round residents. During winter, these residents are largely outnumbered by migratory conspecifics.

Assessing movements and estimating demographic parameters is not straightforward in species/populations with different movement tactics, like partial migrants [1]. This task becomes even more complex in areas where the mixing of non-breeding and breeding partial migration occurs. Yet, such situations are quite common in partial migrants and are typically encountered in areas where the wintering and breeding ranges of a species overlap. The fact that few reliable means are currently available to discriminate sedentary individuals from migrants (a phenomenon called “state uncertainty”), makes it extremely difficult to accurately characterize complex movement patterns [1]. This issue represents a serious challenge for researchers of migration and wildlife managers.

Huge efforts have been devoted worldwide to monitor migratory birds using the ringing of tens of millions of individuals. These ringing schemes have greatly helped avian ecologists to understand timing and patterns of movements [9]–[11], assess population turnover rates [12]–[14], and estimate demographic parameters like survival and site fidelity [15]. Unfortunately, due to spatial heterogeneity in recovery probability ringing data are not well suited to quantify movement patterns. Innovative methods such as isotope ratio analyses, satellite telemetry and geolocators have recently helped improving our knowledge of some aspects of movements including migration processes [16], [17]. However, satellite transmitters are often too heavy to attach to smaller bird species, geolocators do not provide precise geographic position, and both systems can be prohibitively expensive to deploy in large enough sample sizes to achieve sufficient generalization. These methodological constraints, taken together with state uncertainty, have meant that the phenomenon of partial migration and its demographic, ecological or evolutionary consequences have received little attention [1].

Multi-site capture-mark-recapture (CMR) methods which allow taking into account variable ring recovery probabilities may be well adapted to the study of bird movements and the estimation of demographic parameters [18]. However, until recently these methods have been unable to deal with state uncertainty. The development of CMR multi-event methods now allows uncertainty to be taken into account in the assessment of individual state [19], but the potential of such models for assessing movements and demographic parameters of partial migrants with *a priori* different migratory statuses remains to be examined.

In this paper, we investigate seasonal probability of presence and estimate demographic parameters of different categories of individuals belonging to a typical partially migratory duck species, the Common pochard (*Aythya ferina*, hereafter pochard), in an area of South-Western Europe (Loire-Atlantique, France). The species breeding range, which extends from North Africa through South-Western Europe and Central Asia, up to Baikal Lake, is delimited by latitudes 35° and 65°. Whereas northern and eastern pochard populations seem to be forced to leave their breeding grounds in winter, those of South-Western Europe can remain at the same sites throughout the year [20]. Wintering and breeding ranges overlap in these areas, and one can assume that both local breeders and winter visitors cohabit during the wintering season. Seasonal movements of individuals breeding in these areas have not been investigated. Whether they migrate or stay on their breeding grounds during winter is therefore unknown. This is unfortunate because the species, which is heavily hunted [21], has experienced a significant decline over the last 30 years [22]. Therefore, from a sustainable management perspective, characterizing movement patterns and estimating demographic parameters of both local breeders and wintering pochards is urgently needed.

Using a combination of ring-recoveries and capture-live recapture data of individuals fitted with nasal saddles, our aims were: (1) to assess the existence of different categories of individuals based on their patterns of seasonal probabilities of presence on the study area (individuals usually breeding locally which can stay year round in the area or leave the area during winter, *versus* winter visitors coming from other breeding sites) and, (2) to estimate their seasonal survival probabilities and site fidelities. By gathering such information, we expected to improve our understanding of local changes in population size. To achieve these goals, we developed multi-event CMR models that tested for the possible presence of different types of migration strategies among individuals that translate into different probabilities of presence on the study area over the seasons.

## Materials and Methods

### Ethics Statement

Pochards were captured and handled in compliance with legal requirements (ONCFS licences delivered to staff involved in captures and ringing). Birds were fitted with nasal saddles in compliance with the permission delivered by the national authority (Centre de Recherche sur la Biologie des Populations d'Oiseaux, Muséum d'Histoire Naturelle, Paris and Arrêté n°2009-014, Préfecture de Paris). Access to the sampling site was allowed by the manager (Fédération des Chasseurs de Loire-Atlantique, FDC44) as part the management plan of the protected area. At the time of the study the sampling area was co-owned by the ONCFS and the Fondation pour la Protection des Habitats de la Faune Sauvage.

### Study area and field methods

**Figure 1:**
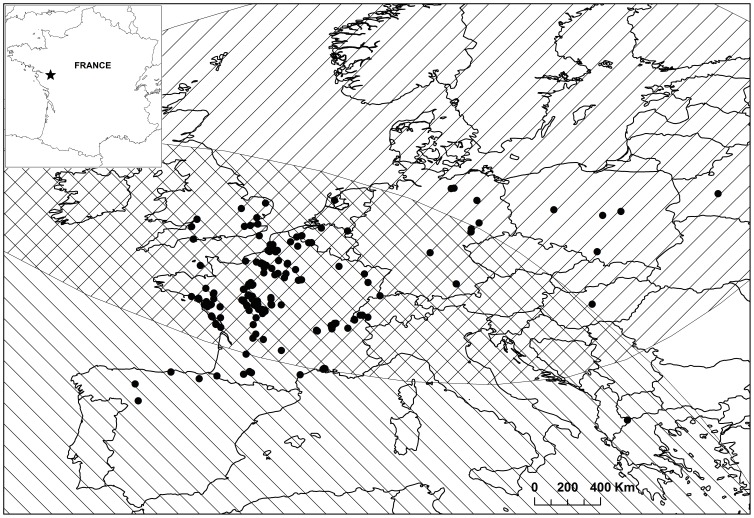
Study area, recapture/recovery locations and species' wintering and breeding ranges. The black star shows the ringing area on Grand-Lieu lake (black star) and the black dots the recapture and recovery locations. Note that the study area is located in the overlapping zone of the breeding (///) and wintering (\\\) ranges of the species.

**Figure 2:**
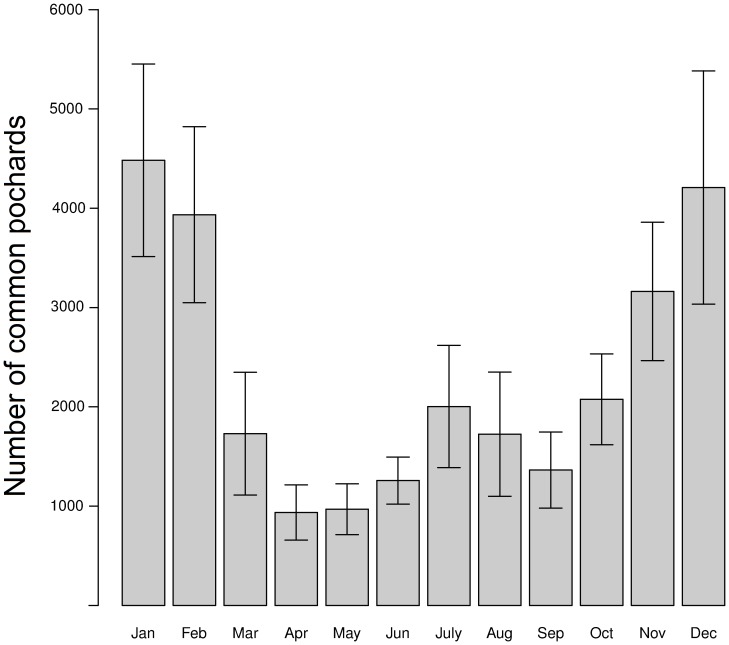
Monthly variations in numbers of pochards on Grand-Lieu lake. Values shown are the averages monthly numbers of individuals (± SD) over the period 2004-2011.

Pochards were captured and marked during both the breeding and wintering seasons on the lake of Grand-Lieu (47° 05′ N, 1° 39′ W) in Western France, from 2004 to 2011 (Fig. 1). Grand-Lieu is considered a major site for waterfowl, including pochards whose winter numbers peak at ca. 5 000 individuals and breeding population averages ca. 550 pairs ([23], Fig. 2). Grand-Lieu is a large endorheic lake of 6 000 ha protected and closed to the public except at the periphery, where marshes representing about 20% of the area are managed for hunting.

During the wintering season, pochards were caught five days a week from early October through March in barrier traps baited with wheat [24]. During the breeding season barriers traps capture sessions occurred from April through early May (before the initiation of incubation). Later in the season, females were also captured on nests using drop-door traps [25]. At the time of capture, each bird was aged as either young (5 to 13 months old) or adult (older than 13 months) and sexed after plumage and cloaca examination [26]. It should be noted that individuals aged as young included only individuals caught from October to their first breeding attempt, and therefore that their birth location was not known. No attempt to ring newly born individuals (<60 days old) of known origin was made (mainly because of the potential risk that could represent the attachment of a nasal saddle for a non-fully grown individual). Each individual was ringed and fitted with a nasal saddle bearing an individual code visible from up to 250 m using a 80×60 spotting scope [27]. Nasal saddle loss was reported for only three out of 600 birds physically recaptured later during the study period, suggesting that this potential problem could be neglected.

Data used in this study included live encounters (resightings and physical recaptures) and ring recoveries collected from April 2004 to January 2011 (Fig. 1). All physical recaptures were performed at the ringing site. Resightings and recoveries were collected throughout the study period by any volunteer observer or hunter anywhere in Europe. Movements of wintering ducks at spatial scales of 10 km are typically considered to be commuting flights between day-roosts and nocturnal foraging areas [28]–[32]. Therefore, all observations or recoveries recorded on Grand-Lieu itself or less than 10 km away were considered under the general “Grand-Lieu” heading, and were distinguished from all other recordings in France and in Europe, pooled as “Elsewhere”.

### Capture-Recapture histories

We divided the annual biological cycle into the breeding season (from 1 April to 31 July) and the wintering season (from 15 November to 15 January), the latter being defined to avoid observations during migration stopovers [20]. Thus, we summarized the 7-year data as capture-recapture histories composed of seven alternating breeding and wintering seasons from breeding 2004 to wintering 2010–2011 (k = 14 occasions). Each capture history started on Grand-Lieu and continued with the subsequent physical recaptures, resightings or recovery either on Grand-Lieu or Elsewhere. Most encounter histories started at the time of the first capture (74% in barrier trap and 13% on nest) and the others started with a live re-encounter (11% physical recapture and 2% resighting) of a bird previously marked on the lake of Grand-Lieu during an inter-season. For a given individual, the data were coded as 0 (not encountered), 1 (capture or resighting on Grand-Lieu), 2 (resighting Elsewhere), 3 (recovery on Grand-Lieu) and 4 (recovery Elsewhere) (Dataset S1). Because only one event could be coded per season, a conflict occurred when two events took place within a single period. Individuals for which dead recovery occurred during the season of ringing were discarded from the analyses (5% of the total number of individuals). As a result our survival rates may be slightly overestimated. An alternative option would have been to assign these dead recoveries to the next capture occasion [33]. However, we did not choose this option because it would have strongly biased our estimates of probabilities of presence which were the focus of the present study. When a dead recovery occurred in the same season as a live reencounter, only the recovery was modeled. When there were several resightings and/or recaptures (events) in different places during the same season, the event on Grand-Lieu was systematically retained rather than choosing randomly among the different events. We opted for this procedure both because it was more informative from a “site manager” perspective and because the site of Grand-Lieu (and the presence of a given individual on it) was the focus of our study. Assessing the presence (even temporary) of individuals of a particular species on a particular site is indeed crucial for implementing adequate management actions. In our study, we were particularly interested in assessing whether or not local breeders were also present (even temporarily) on Grand-Lieu during the wintering season (in order, for example, to assess whether or not local hunting practices during the non-breeding season may negatively affect the breeding population). Nevertheless, this situation which represented only 7% of the total number of events can be considered as relatively marginal.

### Multi-event CMR modelling

Between-individual variations of parameters (heterogeneity) are not accounted for in standard Arnason-Schwartz CMR models. We therefore used the multi-events approach, which is a generalization of CMR multi-site models to uncertain states based upon mixture models with discrete classes of individuals [19], [34], to assess among-individual differences in probabilities of presence (on Grand-Lieu *versus* Elsewhere) and seasonal movements. Multi-event models allow a discrete hidden heterogeneity structure (e.g. between groups of individuals) on the parameters of a multisite CMR model [35] and are therefore well suited for studying individual heterogeneity in movement patterns [36]. In our case, the underlying state occupied by an individual determined its current seasonal location and its probability to emigrate whereas the actual data recorded in the field ("events") corresponded to the current seasonal locations only.

In our study, we expected to detect two main categories of individuals among pochards caught on Grand-Lieu: “local breeders”, i.e. individuals usually breeding on Grand-Lieu which can either be residents or leave Grand-Lieu during winter, as opposed to “winter visitors”, i.e. migratory individuals coming from other breeding sites, present on Grand-Lieu only during winter. Unfortunately these two categories of individuals cannot be distinguished in the field (i.e. when an individual is caught or observed its state/status cannot be determined and is therefore qualified as uncertain). In order to circumvent this problem, we used mixture models to discriminate capture-recaptures histories like "11021" (odd positions in history correspond to breeding seasons), more typical of "local breeders" from histories like "01210", more typical of "winter visitors" (hereafter called type 1 and type 2). Combined with the current location and the status live or dead, this led to the consideration of seven different states: 1) live individual of type 1 on Grand-Lieu "GL^1^", 2) live individual of type 2 on Grand-Lieu "GL^2^", 3) live individual of type 1 Elsewhere "E^1^", 4) live individual of type 2 Elsewhere "E^2^", 5) newly dead individual on Grand-Lieu "ND^GL^", 6) newly dead individual Elsewhere "ND^E^" and 7) the dead absorbing state "D" assigned to individuals that had been dead for more than one capture interval [37], [38]. Newly dead individuals were individuals that died during the ending interval; they are treated separately from long-dead individuals because only newly dead individuals could be recovered.

Multi-event models use three kinds of parameters: the initial state probabilities, the probabilities of transition between the states, and the probabilities of the events conditional on the underlying states. The **initial state probabilities**, i.e. the probability that a newly observed individual was of type 1 or 2, was made dependent on season (breeding *versus* wintering), because the proportion of winter visitors coming from other breeding sites to Grand-Lieu during the breeding season was anticipated to be very low. The transitions probabilities correspond to the movement and survival probabilities. Matrix representations with departure states in rows and arrival states in columns are commonly used. The sum of each row equals one. The first elementary matrix describes the probabilities of movement from the site occupied at occasion *t* (row) to the site occupied at occasion *t+1* (column). We chose to estimate the **probability of presence** on Grand-Lieu at time *t+1* (Ψ^s^_t_) conditional on the site of departure s at time t:
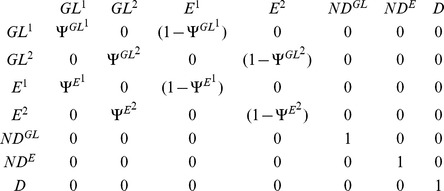
The second elementary matrix was devoted to the **survival probabilities** (Φ^s^_t_) from time t to time t+1 conditional on the sites of departure and arrival:
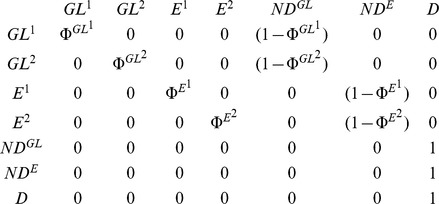
The event probabilities correspond to the probabilities of resighting/recaptures or recoveries on Grand-Lieu and Elsewhere. The third matrix below contains the **recapture probabilities** (*P* ^s^_t_ = the probability for an individual alive at time *t* on site *s* to be physically recaptured or resighted) and the **dead recovery probabilities** (*R^s^_t_* = the probability for the ring of an individual that died on site *s* during the interval between *t* and *t+1* to be recovered):
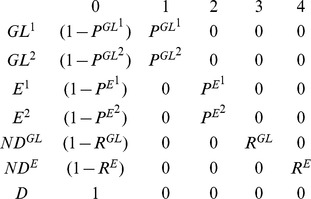
A critical issue in movement studies is that, most of the time, there will be inaccessible and even unknown sites where individuals cannot be tracked. Movements of individuals to unobservable sites and then back to observable sites are defined as temporary emigration [39], [40]. To take into account the possibility of such temporary emigration, we tested the incorporation of an unobservable state as a catchall site for alternative non-monitored sites [41]. This procedure failed because of parameter redundancy and was therefore not included in the following analyses. This is known to happen when temporary emigration is random [41], i.e. when the probability of presence on the monitored sites does not depend on the site occupied at the previous occasion. This seems to be the case here regarding the monitored sites (see results section below) and thus probably also held true for the unmonitored sites. In such a case, probability of presence is confounded with probability of detection but the estimates of the other parameters are unbiased [42].

### Model selection

Probability of presence on Grand-Lieu (Ψ), survival (Φ), recapture probability (*P*) and dead recovery (*R*) were estimated over seasonal intervals (alternating breeding and wintering seasons). The midpoints of the two seasons were 31 May and 15 December. Interval lengths between seasons were therefore 6.5 months from breeding to wintering (31 May–15 December) and 5.5 months from wintering to breeding (15 December–31 May).

Since our data were relatively sparse, we could not fit a very general model, and rather started model selection from a simple standard model which was gradually made more complex (step-up approach). Effects were considered first on recapture/recovery parameters (model selection started by adding effects on these parameters first), then on survival and probability of presence on Grand-Lieu. Model selection included a time/occasion effect (14 successive intervals) on recapture probabilities and dead recoveries. Dead recovery probabilities were allowed to differ according to sites. A site effect (Grand-Lieu *versus* Elsewhere) for recapture probability was also included to account for differences in the type of encounters between the two sites (Grand-Lieu, 79% of physical recaptures and Elsewhere, 100% resightings). We fixed the recapture probability “Elsewhere” to zero during winter 2004–2005 and the breeding season of 2005 because no advertisement on the monitoring program was done towards hunters and observers before these seasons so resightings were very unlikely to be reported to us. Moreover, we included a sex effect on the recapture probability at Grand-Lieu during the breeding season because in addition to baited traps, females were also captured on nests during incubation. Survival probability was modeled as time (13 successive intervals) and age (young *versus* adult) dependent, or as season (31 May–15 December *versus* 15 December–31 May) and age dependent. The probability of presence on Grand-Lieu included season (breeding *versus* wintering) and sex or age effects. In the last step, as an assessment of the presence of winter visitors *versus* local breeders in our sample, we tested whether individuals of type 1 and 2 exhibited differences in their seasonal probability of presence on Grand-Lieu and in survival.

Models were fitted using program E-SURGE version 1.7 [43]. This version allows to take into account unequal interval lengths and consequently to compare monthly survival across seasons. Model selection was performed using the Akaike Information Criterion corrected for small samples sizes (‘AICc’, [44]). In the case of a parameter estimated on the boundary, we used the profile-likelihood interval as its confidence interval [45]. Unless indicated otherwise, results are presented as mean and 95% confidence intervals (CI95%) (see Fig. S1 for examples of design of model constraints in the E-SURGE GEPAT and GEMACO modules).

### Goodness-of-fit tests

Before running our models, we tested whether our dataset did not depart too much from the assumptions of the Arnason-Schwarz (AS) model using goodness-of-fit (GOF) tests implemented in U-CARE [46]. Although the models we fitted make less assumptions than the AS model (they allow for some degree of heterogeneity among individuals that the AS does not authorize) no GOF test is currently available for them [47]. Therefore, we separately examined GOF tests of the live recaptures and of the dead recoveries, as described by Duriez et al. [48] (see Fig. S2 for details on GOF tests).

## Results

A total of 1 161 individuals were marked on the lake of Grand-Lieu over 7 years of study (2004-2011). Among these individuals, 543 were adults (276 females and 267 males) and 618 were young (307 females and 311 males). Most encounter histories (98%) began during the wintering period. Among the 1 161 pochards fitted with a nasal saddle, 254 were later encountered alive at least once on Grand-Lieu (N = 91) or Elsewhere (N = 163). In addition, 14 marked pochards were recovered dead on Grand-Lieu and 23 Elsewhere.

### GOF tests

For physical recapture and resighting data, the goodness-of-fit test was not significant (χ^2^ = 39.98, df = 54, p = 0.92), indicating no difference of fates between newly marked and previously marked individuals (3GSR and 3GSM tests), and no heterogeneity of recapture probability between individuals (MITEC and MLTEC tests) (details and meaning of GOF tests acronyms available in [43]). Similarly, the goodness-of-fit test for an excess of dead recoveries in some seasons, either for both sites taken together (χ^2^ = 16.69, df = 18, p = 0.55) or for each site separately (Grand-Lieu: χ^2^ = 5.2, df = 10, p = 0.88; Elsewhere: χ^2^ = 10.73, df = 11, p = 0.47) were not significant. There was therefore no evidence of non-random temporary emigration.

### Model selection

The best model selected (model 5, Table 1) was one in which the initial state probabilities were dependent on the type of individual and season. According to this model, the recapture probability varied between sites and over time, and the dead recovery probability varied between seasons (breeding *versus* wintering). Monthly survival probabilities also differed between young and adults, as well as between seasons. Interestingly, the model also included a type and a season effect on the probability of presence on Grand-Lieu, suggesting that individuals displaying different seasonal patterns of presence on Grand-Lieu coexisted in our study area. Moreover, the model included a sex effect on the seasonal probability of presence on Grand-Lieu by individuals of type 1 but an age effect during the breeding season by individuals of type 2 (Table 2). This suggests that patterns of presence on Grand-Lieu and therefore movement strategies displayed age and/or gender differences. We considered one other model that was somewhat supported (ΔAICc<2) by the data (model 4, Table1). This model additionally suggested a type and a site effects on the survival probability from the breeding to wintering seasons; however estimates of this model had very large confidence intervals and will therefore not be further discussed.

**Table 1:** Model selection for multi-event CMR models of probability of presence on Grand-Lieu (Ψ) and survival (Φ) of pochards ringed on Grand-Lieu (France).

Model	k	Deviance	AICc	ΔAICc	AICc Weights
**Model 5 Ψ ** ***_sex(type 1,W)+age(type 2,B)+sex(type1,B)_*** ** Φ** _age+season_ P _s*t+sex(B,GL)_ R_season_	**39**	**2893.89**	**2974.04**	**0**	**0.54**
Model 4 Ψ *_sex(type 1,W)+age(type 2,B)+sex(type 1,B)_* Φ_age+season+type *site(B-W)_ P _s*t+sex(B,GL)_ R_season_	42	2888.81	2975.3	1.26	0.29
Model 3 Ψ_s*sex_ Φ_age+season_ P _s*t+sex(B,GL)_ R_season_	34	2961.82	3031.45	57.41	0
Model 2 Ψ Φ_age+season_ P_s*t+sex(B,GL)_ R_season_	31	2985.46	3048.82	74.79	0
Model 1 Ψ Φ P_s*t+sex(B,GL)_ R_season_	29	3018.72	3077.91	103.88	0

Only four intermediate models and the selected one (in bold), among 37 tested, are presented. *P* = recapture probability (including physical recaptures and resightings). *R* = dead recovery probability. Abbreviations include: *age* (adult *versus* young), *season* (breeding *versus* wintering), *s* = site (Grand-Lieu *versus* Elsewhere), *type* (type 1 *versus* type 2), “*” means that the terms acts in interaction while “*+*” that they are additive, B = breeding season, W = wintering season and GL = Grand-Lieu. For example, *sex(B GL) is* a sex effect on Grand-Lieu during the breeding season only; *age(type 2, B) is* an age effect during the breeding season on individuals of type 2 only and type*site (B-W) indicates an interaction between site and type between the breeding and the wintering season. K = number of parameters in the model.

**Table 2:** Seasonal probabilities of presence on Grand-Lieu (Ψ_breeding_, Ψ_wintering_) by pochards according to type (“local breeder” *versus* “winter visitor”), age (young *versus* adult) or sex, derived form the selected model.

		Ψ_breeding_	Ψ_wintering_
**Type 1**	**Females**	**1** (0.70–1)	**0.41** (0.19–0.68)
**“Local breeders”**	**Males**	**0.11** (0.01–0.61)	**0** (0–0.27)
**Type 2**	**Adults**	**0** (0–0.02)	**0.43** (0.26–0.62)
**“Winter visitors”**	**Young**	**0.12** (0.04–0.29)	

95% confidence intervals are in parentheses. Note that while the model objectively retained two types of individuals according to their patterns of presence on Grand-Lieu, the denomination of these types is our own “arbitrary” interpretation of these patterns.

### Initial state probabilities and seasonal probabilities of presence on Grand-Lieu (Ψ)

As predicted, two types of individuals were detected according to their probability of presence on Grand-Lieu over seasons (breeding *versus* wintering): individuals of type 1 whose probability of presence on Grand-Lieu Ψ was higher during the breeding season than during winter *versus* individuals of type 2 for which the reverse was true. The probability that a newly caught individual (initial state probability) was of type 2 (winter visitor) was respectively 0.95 (95%CI: 0.88–0.98) if caught during winter against 0.43 (95%CI: 0.25–0.64) if caught during the breeding season. Due to age and sex effects, four sub-types of individuals could be distinguished according to seasonal differences in probabilities of presence Ψ on Grand-Lieu: 1) females of type 1 whose probability of presence on Grand-Lieu was estimated at 1 (95% CI: 0.70–1) during the breeding season and 0.41 (95% CI: 0.19–0.68) during winter, 2) males of type 1, composed of individuals present during the breeding season with a probability of 0.11 (95% CI: 0.01–0.61) but virtually never present during winter (95% CI: 0–0.27), 3) adults of type 2, composed of individuals present on Grand-Lieu during winter with a probability of 0.43 (95% CI: 0.26–0.62) (probability of presence during the breeding season of 0 (95% CI: 0–0.02)) and, finally 4) young birds of type 2, which included individuals ringed during their first year of life on Grand-Lieu and being present both during their first breeding season and their second winter of life (probability of presence on Grand-Lieu during their first breeding season = 0.12 (95% CI: 0.04–0.29); during their second winter of life = 0.43 (95% CI: 0.26–0.62)).

Therefore two main categories of individuals appeared to be present over the year on our study area: those breeding in the area at least occasionally (hereafter called “local breeders”) and those never seen in the study area during the breeding season (“winter visitors”) coming from other breeding sites.

### Survival probabilities

Adults displayed a higher monthly survival probability than young. Moreover monthly survival was lower between the wintering and the breeding seasons (15 December–31 May) than between the breeding and wintering seasons (31 May–15 December) for both age classes (Table 3). Seasonal survival rates derived from monthly estimates were 0.984 (95% CI: 0.977–0.990) *versus* 0.975 (95% CI: 0.960–0.985) for adult and young, respectively, between the breeding and the wintering seasons, against 0.70 (95% CI: 0.635–0.758) *versus* 0.57 (95% CI: 0.449–0.681) between the wintering and the breeding seasons. Thus, annual apparent survival probabilities were respectively 0.69 (95% CI: 0.623–0.748) and 0.557 (95% CI: 0.433–0.669) for adults and young (computation details in Fig. S3). It should be noted that the annual survival probabilities of young presented here were computed from their first to their second wintering season (from the ages of 4–5 to 16–17 months), and are therefore not necessarily directly comparable to those usually presented in previous studies (e.g. [15]).

**Table 3:** Monthly survival probabilities of pochards ringed on Grand-Lieu in both the wintering and the breeding seasons according to age (young *versus* adult) and season (15 December–31 May *versus* 31 May–15 December).

	15 December–31 May	31 May–15 December
**Adult**	0.937 (0.921–0.951)	0.998 (0.996–0.998)
**Young**	0.904 (0.865–0.932)	0.996 (0.994–0.998)

Values derived form the selected model with 95% confidence intervals in parentheses.

### Seasonal fidelities to Grand-Lieu

Inter-annual fidelities to the breeding and wintering sites were derived from the seasonal probabilities of presence on Grand-Lieu (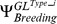
and 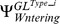). We define “breeding site fidelity” of individual of type i to Grand-Lieu (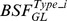) as the probability for an individual breeding on Grand-Lieu a given breeding season to breed on Grand-Lieu the next breeding season (assuming it has survived the interval between both breeding seasons). Similarly, ”wintering site fidelity” of individuals of type i to Grand-Lieu (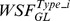) is the probability for an individual wintering on Grand-Lieu a given winter to be present on Grand-Lieu the next winter (provided it has survived the interval between both winters).

Because we are interested in the seasonal fidelities of individuals which will survive the interval between intervening wintering or breeding seasons, fidelity to Grand-Lieu a given season can be estimated as the sum of the product of seasonal probability of presence on Grand-Lieu for individuals staying all year round on Grand-Lieu and for individuals temporarily leaving it in the intervening season (i.e. leaving Grand-Lieu in winter between two breeding seasons or leaving Grand-Lieu during the breeding season between two wintering seasons). A particularity of our study was that the probability to come back to Grand-Lieu from one season to the next was independent of the site occupied during the intervening season (i.e. did not differ between individuals that were present on Grand-Lieu in the previous season and those that were Elsewhere). Consequently, seasonal site fidelity to Grand-Lieu could algebraically be simplified to the probability of presence on Grand-Lieu, that is 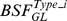 = 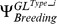 and 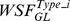 = 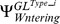 (Table 2). Therefore, the fidelity of females breeding on Grand-Lieu (type 1 or locally breeding females) was 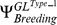 = 1 (see Table 2) whereas their wintering fidelity to Grand-Lieu was equal to 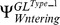 = 0.41. Similarly male of type 1 (“local breeders”) had a BSF of 0.11 and a WSF of 0. Among local breeders both the breeding and wintering site fidelities to Grand-Lieu were thus much higher for females than for males (see Table 2). On the contrary, there was no evidence of a sex difference in wintering site fidelity (WSF) among individuals of type 2 (“winter visitors”, Table 2).

## Discussion

The use of the multi-event approach as a means to overcome our inability to assess *a priori* the migratory status of individuals proved very efficient to identify two types of individuals which were expected to dwell in our study area. The first type included “locally breeding” females whose probability of being present on our ringing site during the breeding season approached 1 and which had a high probability of also being present within the same area during winter (0.41). These females may have a similar probability of presence in winter or be a mixture of three categories of individuals: strict sedentary, strict migrants and facultative migrants (females migrating some years but not others) [49]. Males of this type did not display any of the movement strategies found in females; instead, they seemed prone to switch sites both within and between years. This high site switching propensity is presumed to result from the fact that mating occurs during the wintering season [50] and that, once paired, males follow females to their breeding ground. Indirect evidence for this phenomenon has been gathered in European teal (*Anas crecca*) in which flyway switching was observed more frequently in males than in females [51].

The second type of individuals included adults whose probability of being present in our study area during winter was 0.43 but which were virtually never present within the same area during the breeding season. These birds could therefore be considered as winter visitors (strict migrants) coming from other breeding sites. This type of individuals also included young for which the probability of being present during the wintering season was the same as in adults (0.43). These young birds which could possibly be present in the study area during the first breeding season (0.11) apparently changed behaviour as adults, becoming strict winter visitors. We do not know however if these young birds really attempted to breed, and therefore could be considered as breeding dispersers, or alternatively tended to undertake their pre-nuptial migration later than adults and therefore could be observed on Grand-Lieu at the beginning of the breeding season. Moreover, some young could not have access to the breeding status during their first year of life as has been documented in several species, thereby forming a “floating” non-breeding population on the breeding grounds [52]. Unfortunately our dataset did not allow assessing theses competing hypotheses.

### Seasonal fidelity to Grand-Lieu

Our results concerning site fidelity are consistent with those of the literature on ducks, showing that females whose breeding success highly depends on finding a suitable breeding site are usually much more faithful to their breeding site from one season to the next than males which tend to follow their mate to her breeding ground [53]. The much lower breeding site fidelity in males than in females supports a male- rather than female-mediated gene flow between populations as evidenced in a recent genetic study of this species [54]. Overall, studies of winter fidelity are still scarce in ducks and the results are not directly comparable due to heterogeneity in the spatial scales considered and to the method of estimation of fidelity [55]. Both dabbling and diving ducks appear to display lower fidelity to their wintering site than sea ducks, geese or swans [55]. Interestingly, if we except locally breeding males whose wintering site location was not known, all individuals, whatever age or sex, displayed more or less the same fidelity to Grand-Lieu during the wintering season, with a probability of coming back the next wintering season between 0.41 and 0.43. Our results therefore indicate a higher fidelity rate than previously estimated for the guild of diving ducks (between 0 and 0.2) [55] and comparable to those obtained through the use of nasal saddled individuals in Teal [9]. Food availability (through bait) at the ringing site could account for this relatively high site fidelity and determine how long birds stay in the area during winter.

### Survival patterns

Animals face variable mortality risks over their annual cycle. Changes in mortality risk associated with activities such as reproduction or migration are known to shape the evolution of basic life-history traits. Knowledge of how survival rate varies on a seasonal basis throughout an animal's annual cycle is therefore crucial [56]. We detected lower annual survival rates for young birds than for adults (0.69 for adults and 0.56 for young), with estimates comparable to those computed by [15] for pochards of the same flyway (0.65 for adults and 0.55 for young of about the same age). However, these survival values are probably overestimated because some individual histories had to be removed from our sample in order to avoid biasing our presence/movement and fidelity probabilities. Nevertheless, we gathered evidence for significant seasonal variation in survival, with lower survival rates during late winter/early spring than during summer-early winter. Because hunting is probably the main cause of mortality in this species [21], our results suggest either that hunting pressure was higher after the 15 January than before this date, or that pochards were more vulnerable during the later part of the winter/early spring (e.g. due to courtship, pre- nuptial migration, decrease in food availability, etc.). Late winter is also the period of the year when the most adverse weather conditions are generally recorded in Europe, with the most severe cold spells being registered in January and February. Therefore, adverse weather conditions [15], [57] in interaction with food shortage [58] and hunting pressure could explain the reduced survival probability during late winter/early spring [59].

### Demographic implications

One of the major outcomes of a proper description of the seasonal probabilities of presence of individuals on a given site, such as the one presented in this paper, is its contribution towards a better understanding of seasonal changes in population size and structure. Estimates from the best model suggest that around 95% of the newly marked individuals in winter would be of type 2, that is winter visitors, and therefore that local breeders (type 1 individuals) would form a small part of the overwintering population. Although the newly marked may not be representative of the whole wintering population, the picture they give for the proportion of newly ringed local breeders on Grand-Lieu during winter is compatible with estimates of breeding and wintering numbers. Indeed, while the average volume of pochards wintering on Grand-Lieu would be 7700 [13], the breeding population of females (of which only 41% would be resident in winter) would hardly reach 500 [60]. According to our findings, males breeding on Grand-Lieu would only rarely reside on the site during winter.

Adaptive management requires assessing where individuals are being produced and where they are subsequently being harvested [17]. Even though locally breeding females accounted for a relatively small proportion of the wintering population they displayed a high probability of residence (41% were present at least temporarily in winter), suggesting that a local management schedule specifically designed for the wintering season could have an impact on the long term survival of the “breeding population” of Grand-Lieu. For example, the protection of a part of the site from hunting in winter, as is currently practiced, may indeed help maintain or increase the number of local breeders. Delaying the beginning of the hunting season has been found to benefit locally breeding females in sedentary Portuguese mallard populations [61]. Our results suggest that late winter-early spring is the period of highest mortality in pochards of Grand-Lieu, meaning that management efforts should focus on this period of the year instead of the beginning of the hunting season.

### Towards a better understanding of the underlying factors of partial migration

Although our study was not specifically designed to address questions underlying the ecology/evolution of migratory behaviour, it offers some insights and provides a framework for the development of studies aiming at understanding the determinants of migration [3]. Our most relevant finding in this respect is the difference in migratory behaviour between locally breeding females and males. While sizable proportion of the breeding females winter locally, virtually no males do so. It can also be noted that males, as a general rule, are not very faithful to the breeding site since only 11% of them come back the next year. The fact that females display a high propensity to stay on or close to their breeding site may signify that an intense competition for suitable nesting territories takes place (see [62], [63]). In contrast, males which compete for females during winter [64] would gain little (if anything) from staying on or close to Grand-Lieu during winter. Unfortunately, although we suspect that competition for nesting territories takes place on Grand-Lieu [65], its consequences in terms of migratory behaviour and breeding success have not thoroughly been addressed yet.

## Conclusions and Perspectives

To conclude, the present study is to our knowledge one of the first to give a comprehensive view of among-individual differences in the seasonal probability of presence in a partially migratory species in an area where winter visitors and local breeders potentially mix up during the wintering season. It not only provides a tangible example of the potential of multi-event models for assessing movements, individual state/status (resident *versus* migrant), site fidelity and survival simultaneously, but also shows how helpful such an approach can be for improving our understanding of population dynamics and for studying the evolution of partial migration. Neither movement distances nor the origin or the destination of individuals (especially young ones) could be assessed. This precluded the assessment of key issues like the costs and benefits of movements (a prerequisite towards a comprehensive understanding of evolutionary and ecological processes underlying partial migration), or the relative contribution of local recruitment and immigration to the maintenance of the local reproduction (a key issue of management/conservation). Future work, therefore, should focus on these two aspects in priority and include individual data on reproductive output/success as well.

## Supporting Information

Figure S1 Example of definition of model constraints in E-SURGE using the GEPAT and GEMACO modules.
(PDF)

Figure S2 Goodness-of-fit tests in multi-events models.
(PDF)

Figure S3 Computations of seasonal and annual survival rates using E-SURGE outputs.
(PDF)

Dataset S1 Matrix of the individual capture-mark-recaptures/recoveries histories of pochards caught on Grand-Lieu as formatted for the multi-event approach adopted in our study.
(TXT)
