# Supplementary material for: Movement Patterns in a Partial Migrant: A Multi-Event Capture-Recapture Approach
Source: PLoS One. 2014 May 6;9(5):e96478. doi: 10.1371/journal.pone.0096478 (PMC4011787; doi:10.1371/journal.pone.0096478)
Supplement: Figure S1 — Example of definition of model constraints in E-SURGE using the GEPAT and GEMACO modules. (PDF) [file pone.0096478.s001.pdf]

Figure S1: **Examples of GEPAT/GEMACO instructions on E-SURGE.**

In addition to the matrix description, we provide here details of the practical implementation for the best model in the form of the GEPAT/GEMACO instructions. These are the interfaces of E-SURGE that are used to constrain multi-event models (Choquet *et al.* 2009).
In the model definition (MDL) of E-SURGE "," distinguish two categories, ":" lumps two categories; "." means interactions; "+" refers to additive effects; "&" is used to constrain two parameters to be identical. "f" (from) is the effect of state of departure; "t" is for time effect as a discrete effect; "a" is for age effect (actually "time since first capture") and "g" is for group effect (group 1 is adult females captured, group 2 is young females captured during their first wintering, group 3 is young females captured during their first breeding season, group 4 is adult males captured, group 5 is young males captured during their first wintering, group 6 is young males captured during their first breeding season). Shortcuts can be used to associate a definition to a given name in order to simplify the formulas.
We used the shortcut "adult" defined as
[g(1)&g(4)&g(2).a(3:13)&g(3).a(2:13)&g(5).a(3:13)&g(6).a(2:13)]
We used the shortcut "young" defined as
[g(2).a(1:2)&g(3).a(1)&g(5).a(1:2)&g(6).a(1)]

1. The modeling of the initial state is done through GEPAT and GEMACO as follow:

GEPAT instruction:

p	*	-	-	-	-

GEPAT instruction:
t(1:2:13,2:2:14)

2. The modeling of the movement-transition probabilities (Ψ^{s}_{t}) is done through GEPAT and GEMACO as follow:

GEPAT instruction:

y	-	*	-	-	-	-
-	y	-	*	-	-	-
y	-	*	-	-	-	-
-	y	-	*	-	-	-
-	-	-	-	*	-	-
-	-	-	-	-	*	-
-	-	-	-	-	-	*

GEMACO instruction:
f(2 4).t(2:2:12).g(1 2 3,4 5 6)+f(1 3).t(1:2:13)+f(2 4).t(1:2:13).g(1 2 3,4 5 6)+f(1 3).t(2:2:12).[adult+young]

3. The modeling of the survival probabilities (Φ^s_t) is done through GEPAT and GEMACO as follow:

GEPAT instruction:

s	-	-	-	*	-	-
-	s	-	-	*	-	-
-	-	s	-	-	*	-
-	-	-	s	-	*	-
-	-	-	-	-	-	*
-	-	-	-	-	-	*
-	-	-	-	-	-	*

GEMACO instruction:
[adult+young]+t(1:2:13,2:2:12)

4. The modeling of the recapture probabilities (P^s_t) and the dead recovery probabilities (D^s_t *called* R^s_t in the material and method section) is done through GEPAT and GEMACO as follow:

GEPAT instruction:

*	p	-	-	-
*	p	-	-	-
*	-	p	-	-
*	-	p	-	-
*	-	-	d	-
*	-	-	-	d
*	-	-	-	-

GEMACO instruction:
The first occasion of capture (named "firste") is also part of the matrix and was constant and set to 1 since the capture probability equals 1. The next occasions (events of recapture/resighting/recoveries) are specified by "nexte".

firste+nexte.[f(1 2).t(3:2:13).[t+g(1 2 3,4 5 6)]+f(3 4).t(3,5,7,9,11,13)+f(1 2,3 4).t(2,4,6,8,10,12,14)+f(5 6).t(2:2:14,3:2:13)]

Reference

Choquet R, Rouan L, Pradel R (2009) Program E-SURGE: a software application for fitting multievent models. Modeling demographic processes in marked populations: Springer. pp. 845-865.
