# Supplementary material for: Movement Patterns in a Partial Migrant: A Multi-Event Capture-Recapture Approach
Source: PLoS One. 2014 May 6;9(5):e96478. doi: 10.1371/journal.pone.0096478 (PMC4011787; doi:10.1371/journal.pone.0096478)
Supplement: Figure S2 — Goodness-of-fit tests in multi-events models. (PDF) [file pone.0096478.s002.pdf]

Figure S2: Methods for goodness-of fit tests

In multistate modeling, a GOF test exists for the Jolly Move Model (JMV) but no GOF test is available for mixture of recapture and recoveries. We therefore decided to examine separately GOF tests of the live recaptures and of the dead recoveries as described by Duriez *et al.* (2009). The GOF test for multi-state data is divided into two main components that examine different departures from assumptions. The 3GSR and 3GSM tests compare the fates of newly marked and previously marked individuals ("transience effect"). The last two components (the M.ITECT and MLTEC tests) examine whether individuals observed at time t have a different probability of being caught at time t+1 ("trap effect") (Pradel *et al.* 2005). Only two of its four original main components remain: TEST M.ITEC that will detect an excess of immediate recoveries (i.e. in the season following ringing) and TEST MLTEC that will detect an excess of recoveries concentrated in some later seasons.

Because these GOF tests were run for recaptures and recoveries separately, it was not possible to obtain a global GOF test for the complete data set. Consequently, we could not calculate an overdispersion coefficient *c* (the ratio of the χ^2 statistic to its number of degrees of freedom), classically used to account for the lack-of-fit by correcting model selection criteria (Burnham & Anderson 2002).

References

Duriez O, Sæther SA, Ens BJ, Choquet R, Pradel R, et al. (2009) Estimating survival and movements using both live and dead recoveries: a case study of oystercatchers confronted with habitat change. J Appl Ecol 46: 144-153.

Pradel R, Gimenez O, Lebreton J (2005) Principles and interest of GOF tests for multistate capture-recapture models. Anim Biodivers Conserv 28: 189-204.

Burnham KP, Anderson DR (2002) Model selection and multi-model inference: a practical information-theoretic approach. New York: Springer-Verlag.
