# Supplementary material for: Movement Patterns in a Partial Migrant: A Multi-Event Capture-Recapture Approach
Source: PLoS One. 2014 May 6;9(5):e96478. doi: 10.1371/journal.pone.0096478 (PMC4011787; doi:10.1371/journal.pone.0096478)
Supplement: Figure S3 — Computations of seasonal and annual survival rates using E-SURGE outputs. (PDF) [file pone.0096478.s003.pdf]

Figure S3: Calculations of seasonal and annual survivals

Because we have only correlated estimates of the monthly survivals, care must be taken when deriving combined parameters such as seasonal or annual survivals estimates. We relied on the asymptotic maximum likelihood multi-normal distribution of the β parameters related to the monthly survival estimates φ as follows:

$\mathrm{logit}(\varphi_{ab}) = \beta_1$
$\mathrm{logit}(\varphi_{jb}) = \beta_2$
$\mathrm{logit}(\varphi_{aw}) = \beta_1 + \beta_3$
$\mathrm{logit}(\varphi_{jw}) = \beta_2 + \beta_3$

where 'a' is for adults, 'j' is for juveniles, 'b' is for breeding i.e. interval from May to December, and 'w' is for wintering i.e. from December to May. logit is the logistic function : logit(x)=ln(x/(1-x)).

From the outputs of program E-SURGE, we have

$(\beta_1\ \beta_2\ \beta_3) \sim MN(MU, \Sigma)$ with

$MU = \begin{pmatrix} 6.022827248 & 5.556057607 & -3.31673995 \end{pmatrix}$ and

$$\Sigma = \begin{bmatrix} 0.03908785 & 0.02659942 & -0.02848355 \\ 0.02659942 & 0.06179963 & -0.02889009 \\ -0.02848355 & -0.02889009 & 0.03462031 \end{bmatrix}.$$

Using program MATLAB, we drew 1000000 replicates in this multi-normal distribution and for each replicate we calculated the seasonal and annual survivals according to the formulas given in the paper. We then calculated their means and standard deviations, which are reported in the article.
